# Diversity, Equity, Inclusion, and Belonging in Cardiovascular Disease Fellowship Training

**DOI:** 10.14797/mdcvj.1080

**Published:** 2022-06-03

**Authors:** Ingabire Grace Balinda, Nosheen Reza

**Affiliations:** 1 Perelman School of Medicine at the University of Pennsylvania, Philadelphia, Pennsylvania, US

**Keywords:** diversity, equity, inclusion, cardiology, training, education, disparities

## Abstract

Diversity in the healthcare workforce enhances access to care, reduces health disparities, and improves quality of care for underserved populations. Yet there is a paucity of women and underrepresented minority physicians in cardiology training programs, and progress toward achieving a diverse cardiology workforce has been slow. Here we review the merits of diversity in health care, the current landscape of the cardiology workforce, barriers to increasing the proportion of women and underrepresented minority cardiologists, and specific strategies that have been proposed to sustain and enhance diversity in cardiology training programs.

## Introduction

In 2004, the Institute of Medicine issued a landmark report unequivocally stating that diversity in the healthcare workforce is critical to improving the United States (US) healthcare system.^1^ Several studies have subsequently validated this assertion, demonstrating that a diverse healthcare workforce is a key strategy for tackling perennial health disparities.^2,3^ Physician demographics specifically have been shown to impact cardiovascular health outcomes. Physician gender has been shown to impact quality of care,^4,5^ and patient-physician gender concordance has been associated with improved control of cardiovascular disease (CVD) risk factors.^6^ A randomized controlled trial studying the effect of physician-patient racial concordance on preventive services utilization found that Black men sought more cardiovascular preventive services—particularly invasive services—after meeting with Black physicians compared with non-Black physicians and estimated that this intervention could decrease the Black-White male gap in cardiovascular mortality by 19%.^7^

In 2019, the Accreditation Council for Graduate Medical Education (ACGME) updated its Common Program Requirements to reflect an expectation that all training programs promote recruitment and retention of a diverse workforce.^8^ Given the jarring disparities in CVD treatment and outcomes in racial/ethnic minority populations and women,^9,10,11^ the need to rapidly diversify the cardiovascular clinical workforce could not be more pressing. In this review, we discuss the current climate of diversity in CVD fellowship training, outline the barriers to improving diversity, equity, inclusion, and belonging (DEIB), and summarize strategies to enhance DEIB in training programs. For the purposes of this review, we focus on women and underrepresented minorities as traditionally defined in graduate medical education.

## The Case for Diversity, Equity, Inclusion, and Belonging in the Cardiovascular Workforce

Capers et al.^12^ recently explained the evidence base supporting the merits of a diverse cardiovascular workforce. These benefits included (1) *improved clinical care*, as minoritized individuals are more likely to receive standard of care and follow medical advice when treated by a racially/ethnically concordant physician due to better communication, trust, and reduced implicit bias; (2) *greater access to care in underserved communities*, as underrepresented minority (URM)^13^ physicians more often choose to care for historically excluded populations; (3) *enhanced cultural competence*, as non-URM physicians working in diverse settings become better equipped to care for diverse populations; and (4) *improved research quality*, as diverse research teams broaden the scope of research questions, priorities, and participant enrollment.

### Current Landscape of the Cardiovascular Trainee Workforce

Despite extensively documented benefits of a diverse healthcare workforce, women and URM physicians remain underrepresented in cardiology.^14,15,16^ The percentage of women matriculating in medical school and internal medicine residency has steadily increased over the past decades, and in 2019, women comprised over 50% of US medical students for the first time.^17^ Despite this, cardiology continues to suffer from the “residency-to-fellowship cliff” as the specialty has seen the slowest rate of increase in women trainees over time and continues to be the least diverse internal medicine specialty.^16,18^ Only 24% of US cardiovascular disease fellows are women, with even lower proportions in procedural cardiology subspecialties—14.5% in interventional cardiology and 11% in clinical cardiac electrophysiology.^19^ Over the past 5 years, the percentage of women applying to cardiology fellowships has stalled at around 25%,^18^ and between 2006 and 2018, the proportion of female adult cardiovascular disease fellows only increased from 16% to 21%.^14,20^ In other procedural specialties such as pulmonary and critical care medicine, gastroenterology, and general surgery, women comprise 35%, 38%, and 45% of trainees, respectively.^19^

Unlike the relatively high numbers of women in medical school and internal medicine residencies, URM physicians continue to be underrepresented at every stage of medical training.^14,21^ URM trainees represent 9.9%, 8.9%, 7.4%, and 10.2% of cardiovascular disease, interventional cardiology, clinical cardiac electrophysiology, and advanced heart failure and transplant cardiology fellows, respectively.^22^ In the 5 years prior to 2022, URM trainees have only comprised 9% to 11% of the cardiovascular disease fellowship applicant pool,^19^ and between 2006 and 2016, the percentage of URM cardiovascular disease fellows increased marginally from 11% to 12%.^14^ Comparatively, URM comprise 9%, 10.4%, and 15.4% of pulmonary and critical care medicine, gastroenterology, and general surgery trainees.^22^

Recent research has aimed to uncover drivers of increased diversity in training programs. A 2018 survey of 130 cardiology fellowship program directors found a correlation between program size and trainee diversity, with smaller programs being less likely to have female and URM fellows. All medium and large programs had at least one female fellow; however, 16% of small programs had none. There were no URM fellows in 40% of small programs compared to 30% of medium and 19% of large programs.^15^

Ultimately, these longstanding disparities reverberate across the workforce, with women and URM representing only 13% and 7.5%, respectively, of practicing adult cardiologists,^14^ making the field of cardiovascular medicine poorly representative of the patients whom it serves.

## Barriers to Enhancing Diversity, Equity, Inclusion, and Belonging in Cardiovascular Disease Training Programs

**Figure 1:**
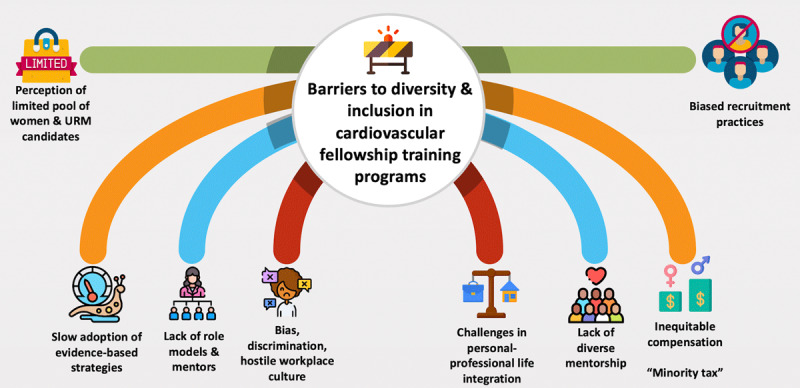
Barriers to sustainable diversity and inclusion in cardiovascular disease training programs. URM: underrepresented minority

Several factors pose a threat to a sustainably diverse and inclusive cardiovascular workforce, including negative perceptions of culture, lack of role models and structured mentorship, biased recruitment practices, and slow adoption of evidence-based merits of diversity at the leadership level, among others (**Figure 1**). A survey of internal medicine residents’ professional development priorities and preferences found that work-life integration, female friendliness, and positive role models ranked highest in professional development needs. Among residents, the strongest perception of the field of cardiology was of a negative culture due to adverse job conditions, interference with family life, and lack of diversity. These factors influenced career choice decisions: women and residents who did not choose careers in cardiology placed more value on work-life balance and had more negative perceptions of cardiology than those who pursued cardiology.^23^

Women who choose to pursue cardiology contend with inequities in compensation^24^ and access to career advancement opportunities^25^ and inadequate support for childbearing responsibilities.^26^ Although the majority of women cardiologists become pregnant during fellowship,^26^ a recent survey found that a sizeable portion of fellowship program directors are not aware of the amount of paid and nonpaid parental leave available to their fellows.^27^ Although the American Board of Internal Medicine allows up to 4 months of leave at the discretion of the program director, 65% of surveyed program directors responded that fellows who utilize all the available leave time should not be eligible for board certification.^27^ The American College of Cardiology’s (ACC) Women in Cardiology Section highlighted several of these barriers through a survey of female cardiologists, which demonstrated that over half of respondents felt pressure to take shorter maternity leave than was available—60% among fellow respondents—and only 46% reported breastfeeding beyond 6 months. Highly cited barriers to breastfeeding included inadequate breaks during clinical practice and lack of appropriate pumping space.^26^ For women interested in pursuing procedural cardiology subspecialties, factors such as lack of female role models, radiation exposure during childbearing years, sexual discrimination and harassment concerns, and the “old boys’ club” culture are additional deterrents.^28,29^

The dearth of URMs entering the physician pipeline continues to be one of the most important barriers to diversifying cardiovascular medicine, owing to a combination of structural racism; lack of adequate educational resources, role models, and mentorship; and bias and discrimination at all stages of education.^30,31^ In a survey regarding barriers to increasing diversity in cardiology training programs, program directors identified the lack of “qualified candidates” and the overall culture of cardiology as the most significant barriers, followed by the lack of diversity in faculty and the program’s surrounding community.^32^ Although much is made of the URM pipeline problem, this does not fully explain the underrepresentation of URM physicians in cardiovascular medicine and medicine in general.

The hostile training and workplace environments that URM physicians experience is another significant barrier to recruitment, retention, and advancement. Studies investigating training experiences of URM residents found that many experience harsher consequences for mistakes, daily microaggressions, bias, and discrimination. Additionally, they are burdened with leading and promoting institutional diversity, feel pressured to conceal parts of their racial/ethnic identity, and experience social isolation.^33,34^ Resource limitation is also a reported obstacle to implementing and sustaining DEIB, especially in smaller, non–university-based programs. Compared to larger and university-based programs, smaller cardiovascular training programs are less diverse and have fewer institutional support mechanisms for URM and female fellows, peer support groups, and formal mentorship/sponsorship programs.^32^

Finally, the lack of prioritizing diversity, inclusion, and equity by the workforce’s gatekeepers is a formidable barrier. Surprisingly, 31% of surveyed cardiology fellowship program directors were uncertain or did not believe that diversity improves quality of care. Only 6% considered diversity among the top three priorities when ranking fellowship applicants, and the majority of those who believed in the merits of diversity could not provide evidence supporting the benefits of a diverse and inclusive workforce.^35^

## Strategies to Improve and Sustain Diversity, Equity, Inclusion, and Belonging in Cvd Training Programs

**Figure 2:**
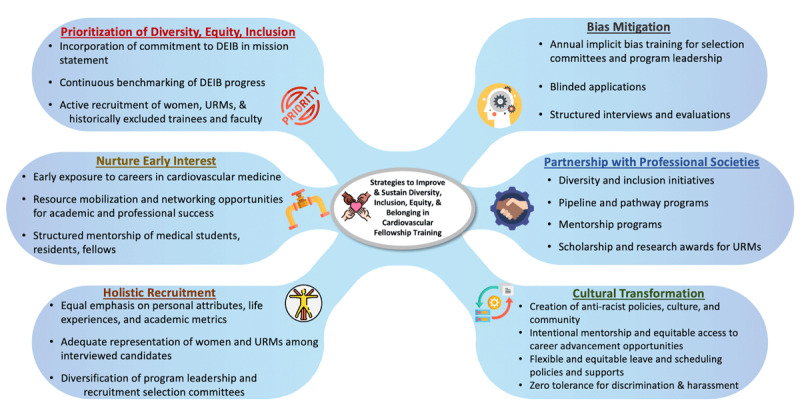
Strategies to improve and sustain diversity, inclusion, equity, and belonging in cardiovascular fellowship training. DEIB: diversity, equity, inclusion, belonging; URM: underrepresented minority; ACC: American College of Cardiology; ABC: Association of Black Cardiologists; AHA: American Heart Association

A multipronged approach is critical to enhance and sustain DEIB in cardiovascular medicine, summarized here in six major strategies: (1) prioritization of DEIB by leadership; (2) holistic applicant review; (3) implicit bias training of gatekeepers; (4) investment in pipeline and pathway programs; (5) cultural transformation and creation of support systems for women and URMs to enhance retention and advancement; and (6) leveraging resources from other key stakeholders (**Figure 2**).

Other procedural specialties with underrepresented women and URM physicians endorse similar strategies, including the American College of Surgeons.^36^ A systematic review of studies describing several diversity and inclusion initiatives in surgical training programs found that holistic review was one of the most effective tools for attracting URM and women trainees into surgery.^37^ Highlighting diversity initiatives on a program’s website also increased the number of women matriculating into surgery.^37^ Small training programs with limited resources can consider low-cost strategies such as explicitly committing to uphold DEIB principles on public platforms such as websites and social media accounts, holistic applicant review and implicit bias training for admissions committee members, and leveraging existing public resources from professional societies. Still, a knowledge gap remains concerning the feasibility of these strategies in smaller programs, and study of dissemination and implementation of successful DEIB initiatives in these settings is needed.

**1. Diversity, Equity, Inclusion, and Belonging as a Top Priority**

To attract and retain women and URMs, programs need to prioritize DEIB by incorporating it into their guiding principles. Suggested practices include reframing mission statements to emphasize commitment to DEIB as a priority. Cardiovascular divisions and departments should endeavor to achieve a trainee and faculty composition that reflects national demographics and to establish and regularly monitor metrics to track institutional progress in incorporating DEIB.^38^ Programs should intentionally seek high-performing URM candidates by recruiting at minority student-run organization meetings and residency fairs at historically Black medical schools and by openly addressing their commitment to and successes and challenges in DEIB on websites, social media, and other outreach platforms.^38^

**2. Holistic Candidate Application Review**

Racial bias in medical student clinical performance evaluations, standardized tests, and election to honor societies has been repeatedly demonstrated. To counter these exclusionary practices, holistic review strategies that incorporate applicants’ nontraditional metrics such as life experiences and personal attributes are being increasingly emphasized in the medical education community.^12,30,38,39^ Such strategies have been shown to enhance diversity in medical training without impacting traditional measures of applicant quality. Through a process that incorporated holistic review, the Ohio State University Medical Center was able to increase representation of URM cardiology fellows from 0% to 25% over a 5-year period, with no significant differences in United States Medical Licensing Examination test scores between URM and non-URM fellows during this period.^40^

**3. Gatekeeper Implicit Bias Mitigation**

Healthcare professionals are not immune from harboring negative implicit biases against people of color and women,^41^ and these biases negatively impact women and URM applicants in the selection process. A study from the Ohio State University College of Medicine demonstrated that a majority of its admissions committee members exhibited significant racial bias on the White implicit association test (IAT). After learning bias mitigation techniques during the subsequent interview cycle, 48% of committee members were conscious of their bias when interviewing and 21% reported that knowledge of their IAT results impacted their admissions decisions. Since the institution of annual implicit bias reduction workshops for the selection committee, the school has consistently matriculated one of the most broadly diverse student populations in the country.^31,42^

Other bias mitigation strategies include diversification of selection committee members, intentional reflection on individual implicit biases and their impacts, provision of bias mitigation strategies on interview days, structured interviews, and blinded application reviews in which candidates’ photos and academic metrics that are subject to racial bias are concealed from committee members.^31,38^

**4. Investment in Pipeline and Pathway Programs**

Due to the paucity of URMs at every stage of the medical education pipeline,^31^ building pipeline programs complete with mentorship and networking opportunities is paramount for efforts to enhance DEIB in cardiovascular medicine. Duvernoy et al. suggested investing in outreach programs spanning the entire educational curriculum, from grade school to internal medicine residency, to ensure early exposure to careers in cardiovascular medicine and dedicated mentorship for women and URM medical students and residents.^30^

Cardiology professional societies have also accelerated efforts to build pathway programs to bolster early interest in medicine and cardiology. As one example, the ACC provides complimentary society membership to medical students and residents and has created several leadership development programs for women and URMs to connect them with career opportunities, mentors, peer networks, and educational resources, including the Young Scholars Program for high school and college students;^43^ the Internal Medicine Cardiology Programs for women, Black, and Hispanic/Latinx trainees;^44^ and the annual Clinical Trials Research boot camp programs.^45^ Similarly, as another example, the American Heart Association (AHA) provides scholarships to a number of students from Historically Black Universities and Colleges to pursue advanced training in biomedical sciences, and its Supporting Undergraduate Research Experiences (SURE) program funds mentored research in cardiovascular medicine for URM students across several academic institutions.^46^ The Association of Black Cardiologists (ABC) has a long track record of early and longitudinal mentorship and sponsorship for its early career members. Other professional societies also are accelerating their structured efforts in this arena.

**5. Cultural Transformation and Creation of Support Systems for Women and URMs**

Efforts to enhance DEIB in cardiovascular medicine will not succeed without a concurrent cultural transformation to meet the unique challenges faced by women and URM physicians. To enhance retention and career advancement of women cardiologists, Sharma et al. advocate for structured mentorship and sponsorship programs to ensure equitable access to research grants, promotion, leadership roles, and pay parity; creative promotion pathways that do not penalize women for childbearing responsibilities; flexible training pathways that optimize work-life integration; adoption of zero tolerance policies for workplace harassment and discrimination; and transparent compensation policies.^47^ Provision of transparent and flexible leave policies to accommodate unforeseen pregnancy complications, radiation safety training, adequate breaks, and private spaces to facilitate lactation will contribute to a culture of inclusion and support for cardiologists with parenting responsibilities.^26^

Efforts to enhance retention of URM physicians will need to tackle implicit and explicit discrimination, professional isolation, lack of mentorship and sponsorship, and the “minority tax” that URM physicians face.^48^ To this end, cardiovascular education leaders have suggested building an antiracist culture in cardiovascular fellowship training by formalizing education on systemic racism, health disparities, and social determinants of health; establishing implicit bias training and standardized mechanisms for addressing and reporting discrimination and microaggressions; actively mentoring and extending scholarly opportunities to URM physicians; ensuring shared responsibility for diversity and inclusion efforts across the program and rewarding these efforts through promotion and advancement; and seeking to understand URM personal experiences with racism and microaggressions through deliberate conversations.^48^

Eberly et al. proposed integrating antiracism practice into the clinical and research training components of the cardiovascular fellowship program through community engagement, funded opportunities for equity-focused quality improvement initiatives, and application of critical race theory to research methodology.^49^

Cardiology fellows are also uniquely positioned to promote a culture of DEIB in their own programs. When leading diverse teams, Njoroge et al. suggest strategies such as setting rotation expectations, facilitating goal-setting, and avoiding competition among trainees; swiftly addressing bias and microaggressions against team members; actively sponsoring trainees by involving them in research projects, connecting them to mentors, providing positive feedback to their training programs; and advocating for a more inclusive culture to program leadership.^50^

**6. Leverage Resources from Other Key Stakeholders**

Many CVD fellowship program directors lack a strategic plan to increase diversity in their programs. Nearly half reported that having access to best practice tools for candidate selection, implicit bias training, parental leave and harassment policies, and guidance with mentoring programs initiation would assist their efforts.^32^ Training programs should leverage external resources from the various professional societies that have committed resources to increasing DEIB in cardiology. For instance, since its inception in 1974, the ABC has provided scholarship and mentorship opportunities to people of color at every stage of the physician pipeline, created fellowship programs in interventional cardiology and clinical cardiac electrophysiology, formed strategic partnerships to enhance investigator diversity in clinical trials, and championed several other diversity and inclusion initiatives across the nation.^51,52^ As another example, gender-specific mentoring in interventional cardiology is available through the Society for Cardiovascular Angiography and Interventions Women in Innovations committee’s mentoring program, aimed at connecting trainees to potential mentors across the country.^53^ The ACC has created resources to guide programs in recruiting a diverse workforce, held workshops to share diversity and inclusion best practices, and provided hands-on training in implicit bias mitigation to program directors.^45^ These resources require widespread dissemination through both formal and informal channels to reach the broader fellowship training community.

## Model Initiatives for Diversity and Inclusion Enhancement Among Cardiovascular Disease Training Programs

Despite widespread recognition of the unique barriers to recruitment and retention of women and URM physicians in cardiovascular medicine, a few fellowship programs have shared successful initiatives aimed at addressing these barriers within their programs. Here, we highlight such initiatives.

### The Ohio State University Medical Center Cardiovascular Disease Fellowship

In 2005, the Ohio State University Medical Center cardiovascular disease fellowship program overhauled its recruitment process to increase its fellowship diversity by (1) setting diversity as a program priority; (2) actively reaching out to external URM residents interested in cardiology; (3) emphasizing mentorship opportunities on interview day; (4) dedicating time for URM applicants to interact with URM fellows and faculty members; and (5) engaging in personalized post-interview communications with competitive applicants to reinforce the program’s interest and commitment to mentorship. Since implementing these interventions, the program has matched URM fellows for 5 consecutive years.^40^

### The University of Pennsylvania Cardiovascular Disease Fellowship

In 2017, the University of Pennsylvania’s female fellows-in-training (FIT) and faculty established the Penn Women in Cardiology (PWIC) program to promote the recruitment, retention, and advancement of women cardiologists. Since its inception, the PWIC has facilitated (1) skill development through targeted workshops; (2) leadership and sponsorship of FITs and early career cardiologists through attendance of national leadership conference and nominations for leadership roles in professional societies; (3) networking through institutional mentoring sessions and meetings with visiting female grand rounds speakers; (4) mentorship pairing of medical students and residents considering careers in cardiology with PWIC members who connect them with clinical and research opportunities and provide guidance through the fellowship application process; and (5) advocacy to enhance recruitment and create supportive workplace environments.^54^

As a result of the PWIC initiatives, several women have served in leadership roles in multiple professional organizations, and an increasing number of women internal medicine residents at the institution have matriculated into cardiology. PWIC also has advocated successfully for increasing dual-physician-trainee parental leave duration from 6 to 12 weeks^54^ and has developed practice guidelines to enable lactating FITs to meet their breastfeeding goals.^55^ These guidelines stipulate that lactating women should have access to well-equipped and appropriately designated clean and private pumping spaces and should be allowed to step away from clinical duties and mandatory engagements at needed intervals for expressing milk. Dissemination of these guidelines across the institution has allowed FITs to better integrate work and parenting responsibilities and created a more supportive environment for fellows growing their families.^55^

### Duke University Medical Center Cardiovascular Disease Fellowship

In 2017, the Duke Cardiovascular Disease fellowship program created multilayered interventions to increase diversity among its fellows, including (1) securing departmental commitment to diversity; (2) formation of a diversity and inclusion task force to devise best practices for recruitment; (3) diversification of program leadership and recruitment committee; (4) restructuring of the application screening process by removing US Medical Licensing Examination score criteria, blinding application reviewers to applicant photos, ensuring women and URM applicants represent at least 25% of those interviewed, and ensuring that all URM applicants are independently reviewed by the URM members of the recruitment committee; (5) restructuring the interview day to maximize interaction between women/URM applicants and potential mentors and colleagues and highlight institutional commitment to diversity; (6) soliciting input from recruitment committee, women, and URM faculty and fellows to ensure diversity in highly ranked candidates; and (7) ensuring structured mentorship for matched women and URM fellows. These interventions resulted in a sustained increase of women and URM fellows matching into the program, more than a doubling compared to the preintervention years.^56^

## Conclusion

There remains an urgent need to diversify the cardiovascular workforce and to simultaneously change workplace culture to promote inclusion, equity, and belonging. These efforts are largely focused at the fellowship level, and adequate education and training at this early stage is necessary to cultivate a representative clinician workforce that is well equipped to care for all patients with cardiovascular disease. Achieving success in these domains will require the intentional reformation of policies, practices, and systems that persistently discriminate against women and URM physicians. Continued benchmarking, systematic assessment of the impact and effectiveness of these strategies, and dissemination of successful strategies will be critical to advance DEIB for the entire cardiovascular training community.

## Key Points

- Despite evidence that healthcare diversity improves care and reduces health disparities, women and underrepresented minority (URM) physicians remain underrepresented in the cardiology workforce.
- The lack of a supportive, inclusive, and equitable culture in the cardiovascular training community continues to thwart diversification efforts.
- Durably enriching the cardiovascular training community will require prioritization of diversity, equity, inclusion, and belonging by academic leaders; investment in pipeline programs providing mentorship and exposing women and URMs to the field of cardiology; holistic recruitment practices; universal implicit bias training with a focus on professional gatekeepers; and eradication of policies, practices, and systems that persistently discriminate against women and URM physicians.
- Systematic monitoring, evaluation, and dissemination of best practices for diversity, equity, inclusion, and belonging will benefit the entire cardiovascular training community.

## CME Credit Opportunity

Houston Methodist is accredited by the Accreditation Council for Continuing Medical Education (ACCME) to provide continuing medical education for physicians.

Houston Methodist designates this Journal-based CME activity for a maximum of *1 AMA PRA Category 1 Credit*™. Physicians should claim only the credit commensurate with the extent of their participation in the activity.

Click to earn CME credit: learn.houstonmethodist.org/MDCVJ-18.3.
